# A Situation Analysis of Parental Awareness Regarding Childhood Hearing Loss to Inform Health Communication Strategy

**DOI:** 10.7759/cureus.113150

**Published:** 2026-07-22

**Authors:** Monika Patel, Shruti Singh, Ujjwal Sourav, Hukam Singh, Rahul Bagla

**Affiliations:** 1 Department of Otorhinolaryngology and Head and Neck Surgery, Government Institute of Medical Sciences, Greater Noida, IND; 2 Department of Community Medicine, Government Institute of Medical Sciences, Greater Noida, IND; 3 Department of Community Medicine, Amrita School of Medical Sciences, Faridabad, IND

**Keywords:** congenital hearing loss, early diagnosis and treatment, knowledge-attitude-practice, newborn hearing, universal newborn hearing screening

## Abstract

Introduction

Early detection through universal newborn hearing screening (UNHS) and timely intervention can improve speech, language, and cognitive outcomes. However, limited awareness among caregivers remains a major barrier to early identification and management. This study performed a situation analysis of parental knowledge, attitudes, and practices (KAP) to identify strategic barriers and inform a targeted health communication strategy.

Methods

A cross-sectional, questionnaire-based study was conducted at a tertiary care hospital in North India from November 2023 to October 2024. A total of 494 participants were recruited. Data were collected through a pre-validated, semi-structured questionnaire administered via face-to-face interviews in Hindi. The tool collected data on knowledge of risk factors and early diagnosis, attitudes toward screening and treatment, and practices related to prevention and care-seeking. Following data collection, a situation analysis and SWOT (strengths, weaknesses, opportunities, and threats) matrix were developed using the Johns Hopkins "P-Process" framework to synthesize raw findings into a strategic roadmap.

Results

The situation analysis revealed a "service-utilization paradox": while 420 (85.0%) of participants supported newborn hearing screening, a profound "follow-up apathy" existed, with 454 (91.9%) incorrectly perceiving subsequent testing as unnecessary. Knowledge of "visible" risks like ear discharge (459, 92.9%) and measles (405, 82.0%) was high, but awareness of "invisible" prenatal and perinatal risks, such as NICU stays and maternal infections, remained poor. The SWOT analysis identified high graduate-level literacy and vaccination compliance (396, 80.2%) as core strengths and opportunities. However, these were countered by a lack of awareness of government facilities (30, 6.1%) and an external threat of outcome pessimism, where fewer than half of the participants believed normal speech development was possible for affected children.

Conclusions

The study underscores a strategic opportunity to leverage high vaccination compliance by integrating hearing health education into immunization clinics. Targeted communication strategies must focus on addressing follow-up apathy and outcome misconceptions to bridge the gap between initial screening and long-term rehabilitation.

## Introduction

With approximately 34 million children worldwide suffering from hearing loss, universal newborn hearing screening (UNHS) has become the standard of care in many countries [1]. Early identification of hearing impairment provides infants with a significantly better chance of developing essential language, speech, and communication skills [1,2]. However, due to resource limitations and a lack of awareness, universal screening does not occur in most parts of the developing world, including India [2,3].

Despite the limited national coverage of UNHS in India, tertiary care centers currently possess the necessary infrastructure and manpower to screen most institutional births [3,4]. Nevertheless, UNHS should not be viewed as a single, isolated test at birth, but rather as a continuous process defined by the "1-3-6" benchmarks: screening by one month of age, confirming a diagnosis by three months, and initiating intervention by six months [5]. Strict adherence to this timeline is essential, as the tangible benefits of UNHS are only realized when initial screening is linked to a reliable system of follow-up and rehabilitation [6].

While current protocols are effective at identifying profound deafness at birth, cases of mild, progressive, or frequency-specific hearing loss are often missed [7]. Ultimately, successfully navigating this clinical pathway requires active caregiver involvement. Therefore, high levels of parental knowledge and positive attitudes toward treatment are critical for reducing the high rates of "loss to follow-up" frequently observed in the Indian context [3,8].

Even in regions where facilities are readily available to screen, diagnose, and intervene during the neonatal period, hearing loss is often detected at a later stage, resulting in significant delays in speech and language development. A primary barrier preventing early intervention is a lack of knowledge and the prevalence of negative attitudes regarding hearing screening, early intervention, and management options among parents [9]. 

A comprehensive situation analysis is therefore essential to understand these behavioral barriers and identify entry points for intervention. The primary objective of this study was to evaluate the baseline knowledge, attitudes, and practices (KAP) of parents and primary caregivers regarding childhood hearing loss. The secondary objective was to utilize these quantitative KAP findings within the structured Johns Hopkins "P-Process" framework [10] to perform a situation and SWOT (strengths, weaknesses, opportunities, and threats) analysis, thereby informing an evidence-based health communication strategy.

## Materials and methods

This cross-sectional, questionnaire-based study was conducted at a tertiary care hospital in North India between December 2023 and October 2024 after obtaining ethical clearance from the Institutional Ethics Committee of Government Institute of Medical Sciences, Greater Noida (GIMS/IEC/HR/2023/41). A purposive sampling approach was employed to recruit parents and primary caregivers of children under the age of five attending the immunization clinic. This setting was selected to capture active caregivers engaged in routine health-seeking behaviors for children, representing the ideal target audience for a community-wide health communication strategy.

The sample size was determined using Cochran’s formula. Assuming a conservative baseline prevalence of 50% for adequate parental awareness, a 5% margin of error, and a 95% confidence interval, the minimum required sample size was calculated as 384. Our final recruitment of 494 participants exceeds this threshold, ensuring adequate statistical power for descriptive and stratified analyses.

A pre-validated, semi-structured questionnaire was used as the primary tool for data collection (an English translation of the questionnaire is provided in the Appendices). The questionnaire was administered in Hindi through a face-to-face interview-based approach to ensure clarity and comprehension across varying literacy levels.

Faculty members and interns from the department of ENT, who were trained beforehand by the team leader, conducted the interviews to ensure consistency and minimize interviewer bias. The training sessions included standardized instructions and mock interviews to develop a robust and uniform interviewing strategy. To maintain data quality, daily reviews were conducted with the team leader, who also regularly audited the completed questionnaires and provided real-time feedback to the interviewers for continual improvement.

The questionnaire was divided into three sections comprising a total of 30 items. The first section collected sociodemographic and socioeconomic information. The second section included 25 close-ended questions (Yes/No/Unsure) aimed at assessing parental knowledge of hearing loss, including its causes, risk factors (such as NICU stay, jaundice, and infections), and the importance of early identification. The final section contained five items designed to evaluate parental attitudes toward interventions for hearing loss, such as hearing aids, cochlear implants, and speech therapy. Participation in the study was entirely voluntary, and written informed consent was obtained from all participants. Anonymity and confidentiality of responses were maintained throughout the data collection process to ensure ethical compliance and encourage honest responses.

Participant responses for each individual KAP item were tracked and analyzed as distinct categorical variables (Yes, No, or Don't Know). Descriptive frequency distributions and percentages were then calculated for each question to pinpoint specific risk-factor misconceptions and behavioral patterns across the study population.

Data were analyzed using SPSS version 26.0 (IBM Corp., Armonk, NY), with a p-value <0.05 considered statistically significant. Descriptive statistics, including means for continuous variables and frequencies or percentages for categorical variables, were used to summarize participant characteristics and KAP responses.

A situation analysis was performed following the Johns Hopkins Center for Communication Programs (CCP) framework [10]. The data from the KAP sections were synthesized to identify the health problem, audience characteristics, and primary behavioral barriers.

A SWOT matrix was developed to categorize internal factors (caregiver willingness) and external environmental factors (availability of government services) influencing early hearing detection and intervention (EHDI).

## Results

Participant demographics and profile

A total of 494 participants were included in the study. The age of the participants ranged from 20 to 68 years, with a significant majority (406, 82.2%) being young adults under the age of 35. The most represented age bracket was 20-30 years, comprising 281 (56.9%) of the sample, followed by 159 (32.2%) in the 31-40 years bracket. Older participants, specifically grandparents, were represented in smaller proportions: 27 (5.5%) were aged 51-60 years and 13 (2.6%) were aged 61-70 years.

Educational attainment was relatively high, with 283 (57.3%) of the sample being graduates. However, a notable portion of the population had limited formal education: 122 (24.7%) reached secondary school, 71 (14.4%) attended primary school, and 18 (3.6%) were illiterate. This profile describes a predominantly young, educated caregiver population supported by a secondary tier of older family members. This is summarized in Table 1.

**Table 1:** Demographic characteristics of the study participants. The percentage was calculated based on the total participants (N = 494). Those older than 40 years are grandparents of the children.

Characteristic	Category	N (%)
Age group (years)	20-30	281 (56.9%)
	31-40	159 (32.2%)
	Older than 40	54 (10.9%)
Educational status	Graduate	283 (57.3%)
	Secondary school	122 (24.7%)
	Primary school	71 (14.4%)
	Illiterate	18 (3.6%)

Knowledge of predisposing factors and risk recognition

Caregivers demonstrated a fragmented understanding of what causes childhood hearing loss, showing a clear divide between the visible postnatal symptoms and invisible prenatal risks.

Awareness was highest for symptomatic causes and infectious diseases. For example, 459 (92.9%) recognized ear pain or discharge as a risk factor, and 405 (82.0%) were aware of the link between measles or mumps and hearing loss. Additionally, 222 (44.9%) identified a delay in the birth cry as a potential indicator of future impairment.

Conversely, knowledge regarding invisible risks was critically low. Only 106 (21.4%) recognized maternal infections during pregnancy as a risk, and just 141 (28.5%) understood the risks associated with consanguinity. Furthermore, only 148 (30.0%) were aware of the dangers posed by prolonged neonatal intensive care unit (NICU) stays (>5 days) or prematurity. These knowledge gaps are detailed in Table 2.

**Table 2:** Knowledge of predisposing and risk factors for childhood hearing loss among study participants. LBW: low birth weight; NICU: neonatal intensive care unit.

Question/Risk factor	Aware/Yes, n (%)	Unaware/No, n (%)	Don't know, n (%)
High fever & maternal infection	106 (21.4%)	339 (68.7%)	49 (9.9%)
Intake of certain medicines	160 (32.4%)	235 (47.6%)	99 (20.0%)
Early and elderly pregnancy	159 (32.2%)	281 (56.9%)	54 (10.9%)
Consanguinity (marriage in relation)	141 (28.5%)	237 (48.0%)	116 (23.5%)
Family history of congenital loss	159 (32.2%)	181 (36.6%)	154 (31.2%)
Delay in birth cry	222 (44.9%)	152 (30.8%)	120 (24.3%)
Neonatal jaundice	211 (42.7%)	267 (54.0%)	16 (3.3%)
Prematurity, LBW, NICU >5 days	148 (30.0%)	212 (42.9%)	134 (27.1%)
Measles and/or mumps	405 (82.0%)	56 (11.3%)	33 (6.7%)
High fever & prolonged hospital stay	99 (20.0%)	241 (48.8%)	154 (31.2%)
Head injury and/or slap to the ear	54 (10.9%)	6 (1.2%)	434 (87.9%)
Ear pain, ear discharge	459 (92.9%)	34 (6.9%)	1 (0.2%)

Knowledge of early diagnosis and the follow-up gap

The analysis of diagnostic awareness revealed a major contradiction between the perceived importance of screening and the necessity of longitudinal care, as shown in Table 2. While 420 (85.0%) of participants acknowledged the importance of hearing screening at birth, a staggering 454 (91.9%) wrongly believed that follow-up after the initial test was unnecessary. Only two (0.4%) of the total 494 participants correctly identified follow-up as a mandatory requirement for continuous monitoring.

Awareness of services and outcomes

General awareness of service availability was poor. Only 30 (6.1%) respondents were aware of existing government facilities or support services. Furthermore, misconceptions regarding outcomes persisted, as only 241 (48.8%) believed that a child with congenital hearing loss could eventually develop normal speech (Table 3).

**Table 3:** Participant awareness regarding early diagnosis, screening protocols, and expected intervention outcomes.

Question	Aware/Yes, n (%)	Not aware/No, n (%)	Don't know, n (%)
Follow-up required after hearing test	2 (0.4%)	454 (91.9%)	38 (7.7%)
Can babies be born with hearing loss?	366 (74.1%)	109 (22.1%)	19 (3.8%)
Can hearing loss be identified at birth?	249 (50.4%)	169 (34.2%)	76 (15.4%)
Importance of hearing screening at birth	420 (85.0%)	74 (15.0%)	0
Was a hearing test done after birth?	263 (53.2%)	127 (25.7%)	104 (21.1%)
Do you suspect hearing loss if the child mispronounces, is dull, or has poor academic performance?	398 (80.6%)	17 (3.4%)	79 (16.0%)
Is there treatment for congenital hearing loss?	267 (54.0%)	26 (5.3%)	201 (40.7%)
Can a child with congenital hearing loss speak normally?	241 (48.8%)	137 (27.7%)	116 (23.5%)
Awareness of government facilities	30 (6.1%)	408 (82.6%)	56 (11.3%)

Caregiver attitudes toward intervention and treatment

Despite significant knowledge gaps, attitudes toward medical intervention were generally positive and proactive (Table 4). A substantial 425 (86.0%) of parents expressed willingness to permit hearing testing immediately after birth. This positive disposition extended to treatment options: 397 (80.4%) were open to their child using a hearing aid, and 398 (80.6%) provided hypothetical consent for ear surgery if diagnosed with a hearing issue. However, 90 (18.2%) remained "unsure" about hearing aids, suggesting a critical need for more targeted counseling.

**Table 4:** Distribution of study subjects according to attitudes toward screening and intervention.

Question	Yes, n (%)	No, n (%)	Don't know, n (%)
Willing to provide permission for hearing test after birth	425 (86.0%)	69 (14.0%)	0
Allow baby to wear hearing aid, if required	397 (80.4%)	7 (1.4%)	90 (18.2%)
Will give attention to ear pain or discharge	403 (81.6%)	91 (18.4%)	0
Consent for ear surgery if diagnosed	398 (80.6%)	81 (16.4%)	15 (3.0%)

Reported ear health and preventive practices

Practices related to general hygiene and preventive care were encouraging, though community engagement remained low (Table 5). Most respondents ensured their children received vaccinations for measles and mumps, represented by 396 (80.2%), and practiced regular ear cleaning, noted by 358 (72.5%). Additionally, 336 (68.0%) reported seeking immediate care for ear infections. Specialized care-seeking was less consistent, as only 303 (61.3%) had ever taken their child to an ENT specialist. Engagement with community education was the lowest-performing metric, with 320 (64.8%) having never attended an awareness program on hearing loss.

**Table 5:** Reported preventive practices and ear health-seeking behaviors among caregivers. ENT: ear, nose, and throat.

Question	Yes, n (%)	No, n (%)	Don't know, n (%)
Do you clean your child's ears regularly?	358 (72.5%)	109 (22.1%)	27 (5.5%)
Have you ever taken your child to an ENT specialist?	303 (61.3%)	155 (31.4%)	36 (7.3%)
Do you avoid exposing your child to loud noise?	373 (75.5%)	101 (20.4%)	20 (4.0%)
Do you seek immediate care for ear infections or discharge?	336 (68.0%)	127 (25.7%)	31 (6.3%)
Have you ensured vaccination for diseases like measles and mumps?	396 (80.2%)	78 (15.8%)	20 (4.0%)
Have you attended any awareness program on hearing loss?	125 (25.3%)	320 (64.8%)	49 (9.9%)

Situation and SWOT analysis

**Figure 1:**
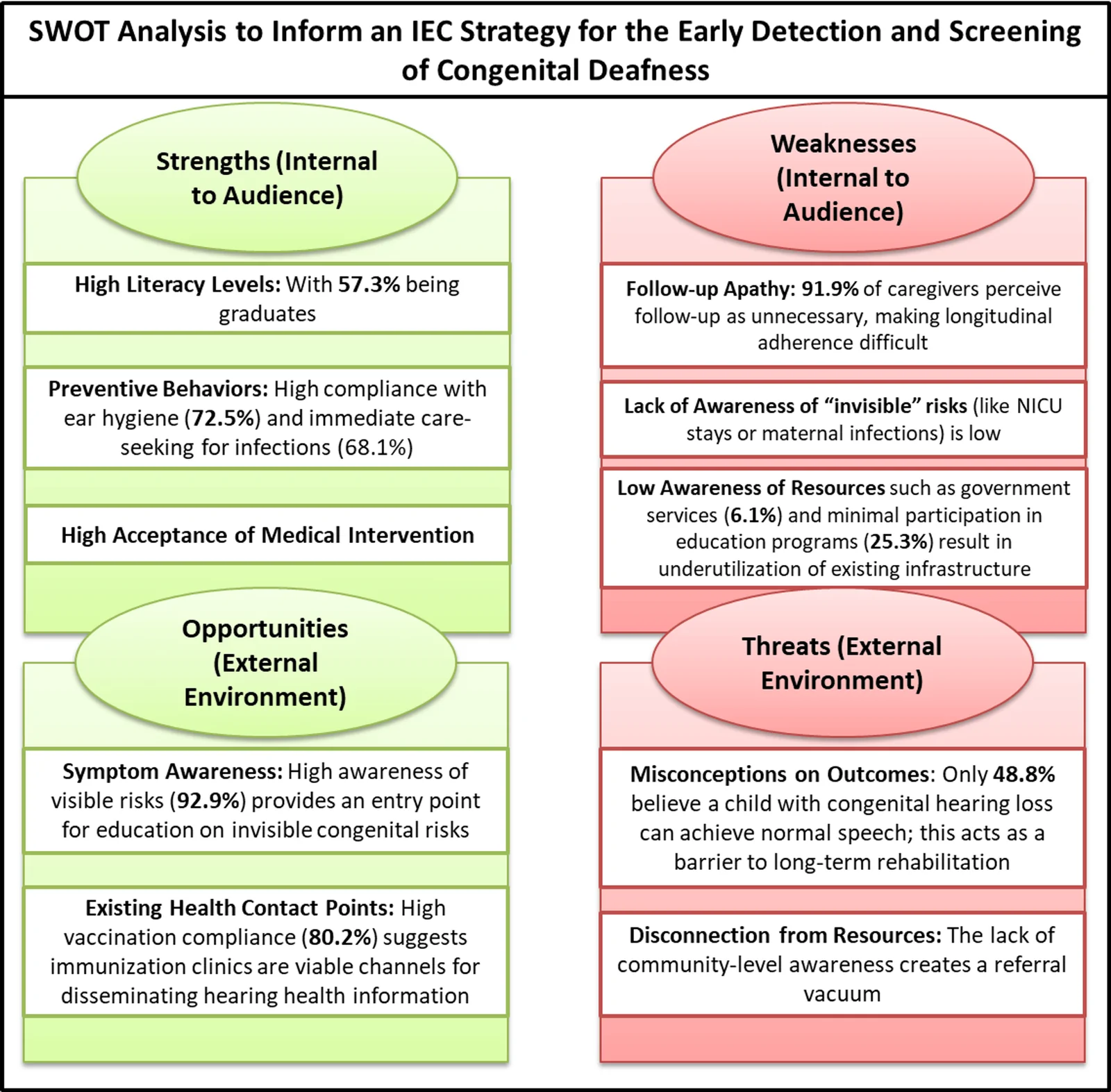
SWOT (strengths, weaknesses, opportunities, and threats) analysis identifying strategic drivers for a childhood hearing health communication strategy. This figure categorizes the internal and external factors influencing early hearing detection and intervention (EHDI) in North India. IEC: information, education, and communication. Image credit: Dr. Shruti Singh.

Following the O’Sullivan et al. (2003) framework [10], the quantitative data were synthesized into a strategic situation summary (Table 6) and a SWOT analysis (Figure 1).

**Table 6:** Situation summary worksheet for childhood hearing loss. A strategic synthesis of the health problem, target audience demographics, and key behavioral challenges, identifying awareness as the primary barrier to service utilization, based on the O’Sullivan et al. (2003) framework [10]. Table credit: Dr. Shruti Singh.

Component	Core findings and inference	
Health problem	Delayed detection and intervention of congenital and childhood hearing loss	
Potential primary audience	Parents (mothers and fathers) and grandparents of children under five years. Demographic: Young adults aged 20-30 years (56.9%) with graduate-level education (57.3%)	
Key challenges	Low risk factor awareness	Maternal infections (21.4%), consanguinity (28.5%), NICU stay over 5 days (30%)
	Low awareness of screening service	Only 6.1% of participants are aware of government facilities; 64.8% of participants have never attended an awareness program on hearing loss
	Low awareness about follow-up	91.9% of caregivers believe follow-up after a hearing test is unnecessary
	Misconceptions on outcomes	Only 48.8% of participants believe a child with congenital hearing loss can speak normally
Key opportunities	Risk factor awareness	Awareness of visible symptoms like ear discharge is high (92.9%)
	Positive attitudes	86% of parents are willing to permit newborn hearing testing, and over 80% support hearing aids and surgery
	Existing practices	High compliance with vaccination (80.2%) and hygiene practices (72.5%)
Reality	The primary barrier is a lack of awareness rather than resource limitations, especially for children born in tertiary care facilities where screening resources are readily available but underutilized or not followed up on	
Expected contribution	Communication interventions can bridge the gap between initial screening and necessary follow-up and increase the utilization of available government services	

The analysis identified high parental willingness for screening and high vaccination compliance as core strengths and opportunities, respectively. Conversely, follow-up apathy and low awareness of government services (6%) were categorized as primary weaknesses and threats. These findings serve as the foundation for the proposed health communication strategy.

## Discussion

The findings of this study move beyond descriptive statistics by utilizing a structured strategic communication framework to transition from raw data to a targeted public health roadmap [10]. The resulting situation analysis (Table 5) identifies a critical "service-utilization paradox": while 85% of parents support newborn screening and over 80.4% are receptive to interventions like hearing aids, a profound "follow-up apathy" exists. A staggering 91.9% of caregivers incorrectly believe that subsequent testing after an initial birth screen is unnecessary, identifying a major behavioral leak in the clinical pathway. This is further supported by recent findings from Alharbi et al. [11], where despite a massive 82.9% favoring hearing services, over 76% of caregivers remained unaware of how hearing loss directly impacts developmental milestones.

This paradox is further elucidated through the SWOT analysis (Figure 1), which identifies that the primary barrier is not a lack of resources, but an informational disconnect. The core strengths of high graduate-level literacy (57.3%) and strong preventive habits, such as high vaccination compliance (80.2%), provide an immediate opportunity to integrate hearing health education into existing high-frequency touchpoints like immunization clinics. However, these are countered by internal weaknesses, specifically a lack of awareness regarding "invisible" risks and government facilities (6.1%). These internal gaps are exacerbated by the primary threat of outcome pessimism, as only 48.8% believe a child with congenital hearing loss can achieve normal speech.

Our findings regarding these knowledge gaps and demographic variabilities are consistent with several real-world studies. There is cultural and regional variability in awareness, with a knowledge gap evident in lesser-known risk factors like consanguinity and NICU admissions, as highlighted in a cross-sectional study by Yoshita et al. [12] involving over 300 parents and caregivers. It was noted that mothers had better awareness than fathers, and a lack of knowledge was more evident in older caregivers, such as grandparents, reinforcing the importance of age- and role-specific educational outreach. Similarly, a study by Mazlan and Dar [13] assessed KAP among 150 parents and found that while overall knowledge was moderate (67.3% showed good awareness), specific awareness for postnatal infectious causes like measles was low. Their study also revealed that mothers were more knowledgeable than fathers, and educational levels significantly influenced awareness levels. The findings of our study are consistent with those of the above-published studies in similar populations; our results align with these findings, as the majority of our respondents were graduates who showed higher awareness of common symptoms but less clarity on congenital or perinatal factors.

Furthermore, Alqudah et al. [14] evaluated family physicians' knowledge and practices in Saudi Arabia, showing strong support for early screening but limited awareness about national hearing loss programs. This reflects the results of the current study, where only 6.1% of respondents were aware of available government facilities. Overall, these comparisons support the need for improved public education, inclusive outreach to all caregivers including grandparents, and better dissemination of available services and interventions for childhood hearing loss.

The findings of this study regarding the varied levels of awareness among participants about risk factors associated with childhood hearing loss are supported by several real-world research studies on KAP among parents and caregivers. Similar to the present findings, in the cross-sectional study conducted by Yoshita et al. [12], the study reported high awareness regarding symptomatic causes such as ear discharge and infectious conditions like measles. However, it also highlighted poor recognition of less visible but critical risk factors such as maternal infections during pregnancy, consanguineous marriages, and neonatal complications. Another study by Mazlan and Dar [13] examined KAP among 150 parents of children under five years. That study found that while 79.3% of parents were aware of congenital causes of hearing loss, only 29.3% recognized postnatal infections such as measles as a contributing factor. This contrasts with the current study, where measles awareness was relatively high (82%). Our study found that the understanding of NICU admission, birth complications, and head trauma as risk factors remained poor. Alenzi et al. [15] also found very similar deficits in Tabuk, specifically citing low awareness of jaundice (21.7%), low birth weight (28.4%), and chemotherapy (38.2%).

Additionally, Alqudah et al. [14] conducted a study among 133 family physicians in Saudi Arabia to evaluate their KAP regarding pediatric hearing loss. While the focus was on medical professionals, the study is relevant because it highlights the indirect effect healthcare providers have on parental awareness. Although most physicians recognized major risk factors like family history and meningitis, only a third were aware of national screening programs, implying that information about hearing loss is not adequately reaching families through health systems. This is a concern mirrored by the low percentage (6.1%) of participants in the current study who were aware of government facilities for hearing-impaired children.

The findings of the present study, which reveal both encouraging levels of awareness and critical knowledge gaps regarding early diagnosis of childhood hearing loss, are consistent with existing literature. In a study by Mazlan and Dar [13], it was reported that while a substantial proportion of parents (around 79%) were aware that hearing loss can be congenital, only 42% knew that it can be identified at birth. Furthermore, only a minority of the participants had actually undergone newborn hearing screening for their children, despite expressing positive attitudes toward early diagnosis. These findings closely mirror those of the present study, where although 74.1% acknowledged that babies can be born with hearing loss and 85% supported screening at birth, only 53.2% reported that their child had undergone such testing. Additionally, the near absence of awareness regarding the need for follow-up (only 0.4% in the current study) was also reflected in the work of Mazlan and Dar [13], where continuity of care and follow-up after initial testing were poorly understood.

Similarly, Yoshita et al. [12] emphasized that while general support for newborn hearing screening was strong, significant misconceptions existed about treatment outcomes and the long-term development of children with hearing impairment. Only half of their respondents believed that children with congenital hearing loss could develop normal speech if treated early, a result that aligns with the 48.8% in the current study who shared the same belief. This reflects ongoing myths and stigma surrounding speech and hearing development despite medical intervention. Furthermore, Alqudah et al. [14] revealed a concerning gap in knowledge about structured follow-up systems and government programs. While most physicians acknowledged the importance of early identification, only about one-third were aware of the national EHDI programs. This supports the current study’s finding that only 6.1% of participants were aware of government-provided support, indicating a need for better communication and integration between healthcare systems and public awareness.

The analysis of participants’ practices related to childhood hearing health demonstrates a pattern that is both promising and concerning. On one hand, the study reveals that most caregivers engage in important day-to-day preventive behaviors such as cleaning their child’s ears (72.5%) and avoiding exposure to loud noise (75.5%), which reflects a basic awareness of factors that can protect against hearing damage. These findings are consistent with other studies conducted in similar contexts, such as that by Yoshita et al. [12], which showed that parents often demonstrated higher compliance with routine hygienic practices than with clinical follow-ups.

Furthermore, the fact that 68% of participants reported seeking immediate care for symptoms like ear discharge or infections is an encouraging indicator of responsiveness to acute conditions. This aligns with observations in the study by Mazlan and Dar [13], where care-seeking behavior was found to be adequate when symptoms were obvious or distressing. Similarly, 80.2% of participants in the current study had ensured vaccination against measles and mumps, demonstrating an indirect but vital measure to prevent hearing loss, particularly considering that measles-related deafness remains a preventable cause of childhood hearing impairment in developing countries.

Despite these positives, gaps in more structured, preventive healthcare practices are evident. Only 61.3% of participants had taken their child to an ENT specialist, indicating that routine evaluations or professional consultations are not yet a norm. This can result in delayed identification of subtle or progressive hearing impairments. In addition, the very low proportion (25.3%) of participants who had ever attended an awareness program on hearing loss, coupled with 64.8% having never participated and 9.9% being unsure, points to a major gap in community-level health education and advocacy. These shortcomings are particularly important in the context of congenital or early-onset hearing loss, which may not be detected through hygiene or symptom-based observation alone. As emphasized by Alqudah et al. [14], community-based hearing health programs, especially those embedded within routine immunization or school health services, can play a crucial role in raising awareness and encouraging professional evaluations even in the absence of overt symptoms.

Study limitations

This study is subject to several limitations that should be considered when interpreting the results. The major limitation is that the study was conducted among caregivers of infants attending a healthcare facility in Uttar Pradesh, which may limit the generalizability of the findings to populations with different cultural or socioeconomic backgrounds. Additionally, because the survey was interview-based, the results may be influenced by social desirability bias. Also, while the sample size was sufficient for descriptive analysis, a larger multicentric study would be beneficial to further validate these findings across diverse healthcare settings. Finally, while the SWOT analysis serves as a highly structured and widely accepted interpretive framework for health communication planning, it functions as a strategic roadmap rather than an independently validated empirical tool.

## Conclusions

This study reveals a critical service-utilization paradox: while there is high parental support for initial newborn screening, a profound "follow-up apathy" exists where the vast majority of caregivers perceive subsequent testing as unnecessary. This identifies a significant behavioral leak in the clinical pathway that is compounded by an external threat of outcome pessimism, as fewer than half of the participants believe that normal speech development is possible for children with congenital hearing loss. These internal weaknesses and external misconceptions highlight that the primary barrier to early intervention is informational and behavioral rather than a lack of physical resources.

While preventive practices are fairly well-adopted, professional consultations and participation in formal awareness programs remain limited. The findings emphasize the strategic opportunity to leverage core strengths, such as high literacy and strong vaccination compliance, by integrating hearing health education into existing high-frequency touchpoints like immunization clinics. Strengthening these community-based interventions is essential to bridge the gap between initial screening and the necessary long-term management of childhood hearing loss.

## Appendices

**Table 7:** English translation of the study questionnaire. UHID: unique health identification; NICU: neonatal intensive care unit. Section A-C: Respondents were asked to select "Yes," "No," or "Not Sure" for each item to identify specific knowledge gaps and misconceptions.

No.	Item	Response/Field
I. Sociodemographic details		
i	Name/Age	
ii	UHID	
iii	Date	
iv	Father's name	
v	Mother's name	
vi	Father's age/Mother's age at birth	
vii	Religion	
viii	Consanguinity (Marriage in relation)	
ix	Parental education (Father/Mother)	
x	Family income	
II. Knowledge		
A	Risk factors for deafness	
i	Can high fever and viral infection in the mother during pregnancy cause hearing loss in the child?	( ) Yes ( ) No ( ) Not Sure
ii	Can the intake of certain medicines during pregnancy cause hearing loss in the child?	( ) Yes ( ) No ( ) Not Sure
iii	Does early or late-age pregnancy lead to a reduction in the child's hearing capacity?	( ) Yes ( ) No ( ) Not Sure
iv	Is there a relationship between consanguineous marriage and hearing loss?	( ) Yes ( ) No ( ) Not Sure
v	Can a family history of congenital hearing loss cause hearing loss in an infant?	( ) Yes ( ) No ( ) Not Sure
vi	Can a delay in the birth cry lead to reduced hearing capacity?	( ) Yes ( ) No ( ) Not Sure
vii	Can neonatal jaundice cause hearing loss?	( ) Yes ( ) No ( ) Not Sure
viii	Can prematurity, low birth weight (<1.75 kg), or a NICU stay >5 days cause hearing loss?	( ) Yes ( ) No ( ) Not Sure
ix	Can postnatal measles and/or mumps cause hearing loss?	( ) Yes ( ) No ( ) Not Sure
x	Can high fever and prolonged hospitalization lead to hearing loss?	( ) Yes ( ) No ( ) Not Sure
xi	Can a head injury and/or a slap to the ear cause hearing loss?	( ) Yes ( ) No ( ) Not Sure
xii	Can ear pain or ear discharge lead to hearing loss?	( ) Yes ( ) No ( ) Not Sure
B	Early diagnosis	
xiii	Is follow-up required after a hearing test?	( ) Yes ( ) No ( ) Not Sure
xiv	Can babies be born with hearing loss?	( ) Yes ( ) No ( ) Not Sure
xv	Can hearing loss be identified at birth?	( ) Yes ( ) No ( ) Not Sure
xvi	Do you think hearing screening at birth is important?	( ) Yes ( ) No ( ) Not Sure
xvii	If a child becomes dull, mispronounces words, or has poor academic performance, do you suspect hearing loss?	( ) Yes ( ) No ( ) Not Sure
C	Early intervention	
xviii	Is there a treatment available for congenital hearing loss?	( ) Yes ( ) No ( ) Not Sure
xix	Do you think a child with congenital hearing loss can speak normally?	( ) Yes ( ) No ( ) Not Sure
xx	Are you aware of facilities/support services provided by the government?	( ) Yes ( ) No ( ) Not Sure
III. Attitude		
xxi	Will you permit hearing screening for your child immediately after birth?	( ) Yes ( ) No ( ) Not Sure
xxii	If required, would you consent to a hearing aid for your child?	( ) Yes ( ) No ( ) Not Sure
xxiii	Should attention be given to ear pain or ear discharge?	( ) Yes ( ) No ( ) Not Sure
xxiv	What would your reaction be if you found out your child has deafness?	
xxv	If required, would you consent to ear surgery for your child?	( ) Yes ( ) No ( ) Not Sure
